# Serum 25-hydroxyvitamin D is not related to cardiac natriuretic peptide in nulliparous and lactating women

**DOI:** 10.1186/1472-6823-9-4

**Published:** 2009-01-29

**Authors:** Hussein F Saadi, M Gary Nicholls, Christopher M Frampton, Sheela Benedict, Javed Yasin

**Affiliations:** 1 Department of Internal Medicine, Faculty of Medicine and Health Sciences, UAE University, Al Ain, United Arab Emirates; 2 Department of Medicine, Christchurch School of Medicine and Health Sciences, Christchurch Hospital, Christchurch, Canterbury, New Zealand

## Abstract

**Background:**

Vitamin D deficiency is associated with heightened risk of cardiovascular disease. Potential mechanisms include involvement of vitamin D in regulation of renin-angiotensin system and manufacture and secretion of cardiac natriuretic peptides. Our aim was to document relationships between 25 hydroxyvitamin [25(OH)D] and N-terminal pro B-type natriuretic peptide (NT-proBNP) and plasma renin activity (PRA) levels and to document the effect of vitamin D administration on NT-proBNP and PRA levels in vitamin D deficient subjects.

**Methods:**

Serum 25(OH)D, parathyroid hormone (PTH), plasma or serum NT-proBNP and PRA levels were measured at baseline in nulliparous and lactating women and after 2 months of oral vitamin D_2 _(2,000 IU/day or 60,000 IU/month) supplementation to lactating women.

**Results:**

Baseline levels of 25(OH)D were low (<50 nmol/L) in most women whereas PRA and NT-proBNP levels were within the normal range. There were no significant correlations between baseline 25(OH)D or PTH with NT-proBNP and PRA. Vitamin D administration over a 2-month period in lactating women was associated with a decline in NT-proBNP (by 9.1 ± 2.0 pmol/L; p < 0.001) and PRA (by 0.32 ± 0.17 nmol/L/hr; p = 0.064). However, there were no significant correlations between the changes from baseline in 25(OH)D and either NT-proBNP (r = -0.04, p = 0.8) or PRA (r = -0.04, p = 0.8).

**Conclusion:**

We found no significant correlations between 25(OH)D or PTH with NT-proBNP and PRA in vitamin D deficient women. Further information is required to clarify the effects of vitamin D administration on cardiac structure and function.

## Background

In addition to metabolic bone disease, new evidence links vitamin D deficiency with many disease states including autoimmune diseases, multiple sclerosis, some forms of cancer (breast, ovarian, colon), metabolic syndrome and type 2 diabetes [1-3]. More recently, vitamin D deficiency has been linked with heightened risk of hypertension and cardiovascular disease [4-8]. Potential mechanisms that may explain this link include involvement of vitamin D in the regulation of the renin-angiotensin system [9-11], and the negative vascular effects of secondary hyperparathyroidism [12]. In addition, activation of nuclear vitamin D receptors present in the myocardium can inhibit cardiac growth and hypertrophy whilst also suppressing the manufacture and secretion of the cardiac natriuretic peptides in both atrial and ventricular myocytes [13].

In some Arab countries, vitamin D deficiency is particularly common especially amongst women [14,15], and it has been proposed that correction of low vitamin D status in such a cohort might reduce the incidence of cardiovascular disease, especially heart failure [16]. Circulating levels of B-type natriuretic peptide (BNP) and the 1–76 amino-terminal fragment of pro-BNP (NT-proBNP) are elevated in acute and chronic cardiac impairment in proportion to the degree of hemodynamic compromise [17,18]. NT-proBNP rises more steeply than pro-BNP as cardiac function deteriorates and may be a more sensitive marker of cardiac dysfunction [18]. It was recently demonstrated that both BNP and NT-proBNP correlated inversely with 25 hydroxyvitamin D [25(OH)D] concentrations (r_s _= -0.60, p = 0.007 and r_s _= -0.64, p = 0.003, respectively) in patients on maintenance peritoneal dialysis [19].

The objectives of the present study were to document relationships between plasma levels of NT-proBNP and serum 25(OH)D concentrations and to document the effect of vitamin D administration on NT-proBNP and plasma renin activity (PRA) levels in vitamin D deficient subjects.

## Methods

The study protocol was approved by the human research ethics committee of the Al Ain Medical District and subjects received both oral and written information and gave informed consent. We recruited 88 generally healthy nulliparous Emirati women in the reproductive age group, many of whom were medical students and interns working at Tawam hospital in Al Ain city, and 90 lactating women (15 UAE, 61 other Arab, and 14 South Asian) at the time of their first postnatal visit to the Maternal and Child Health center in Al Ain city (latitude 24°N and longitude 55°E) [14]. Lactating women were eligible if they planned to continue breast-feeding for the next 3 months. Exclusion criteria included pregnancy, history of metabolic bone disease or calcium disorders and treatment with vitamin D (other than multivitamins) within the past 1 year. Enrolment began in September 2005 and finished in February 2006.

This was an open label randomized parallel group clinical trial of lactating and nulliparous women [14]. Consenting subjects in each group were randomly allocated to either 2000 IU daily dose or 60,000 IU monthly dose of vitamin D_2 _in a 1:1 ratio within permuted blocks of size 10. Of the 88 nulliparous women enrolled initially, 55 completed 1 month, 27 completed 2 months and 23 completed the 3-month study period. Most subjects who withdrew from the study were contacted and none specified any particular reason for withdrawal. Sufficient follow-up serum samples were also not available except for very few subjects. We therefore measured NT-proBNP only in baseline samples from subjects with sufficient stored serum (n = 53) using an electrochemiluminescence immunoassay (Roche Elecsys 1010/2010 system). Intra-assay variability was <1.5%.

Of the 90 lactating women enrolled initially, 69 completed 1 month, 58 completed 2 months and 48 completed the 3-month study period. There were no significant differences in baseline characteristics between women who completed the study and those who dropped out. Venous samples drawn at baseline (21 ± 3.4 days, mean ± SEM, post partum) and monthly thereafter showed peak levels of 25(OH)D at 2 months [14]. We therefore measured NT-proBNP and PRA in baseline and 2 month samples from subjects with sufficient stored plasma (n = 53 subjects for PRA and n = 46 subjects for NT-proBNP). Original samples were taken into chilled tubes containing EDTA and centrifuged at +4°C and the plasma stored at -80°C. One aliquot of plasma from each subject was couriered on dry ice to the Endolab in Christchurch, New Zealand where NT-proBNP and PRA were measured in single assay runs to avoid inter-assay variability [18]. Intra-assay variability varied between 3.7% for PRA and 6.7% for NT-proBNP. Reference ranges were NT-proBNP 2–50 pmol/L, and PRA 0.4–2.3 nmol/L/hr. Serum calcium was measured with Beckman Synchron autoanalyzer. Serum 25(OH)D concentrations were determined by a radioimmunoassay that measures both 25(OH)D_2 _and 25(OH)D_3 _equally (DiaSorin; Stillwater, Minnesota). The intra- and inter-assay coefficients of variation (CVs) were 8.3% and 3.2%, respectively. Serum 25(OH)D concentration <50 nmol/L (20 ng/ml) was considered to reflect vitamin D deficiency based on studies in the literature (19, 25). Serum intact parathyroid hormone (PTH) was measured by immunoradiometric assay (Diagnostic Products Corporation; Los Angeles, California). The intra- and inter-assay coefficients of variation (CVs) were 6% and 5.1%, respectively.

Data were analysed with SPSS statistical software (version 15; SPSS Inc, Chicago). Data are shown as mean ± SEM unless mentioned otherwise. Differences between groups were assessed by two-tailed *t*-tests for continuous variables and by chi-square tests for categorical variables. Paired observations were analysed using paired two-tailed *t*-tests. Correlations between different variables were examined using Spearman's correlation coefficients. P-values < 0.05 were considered significant.

## Results

### Baseline data

**Figure 1:**
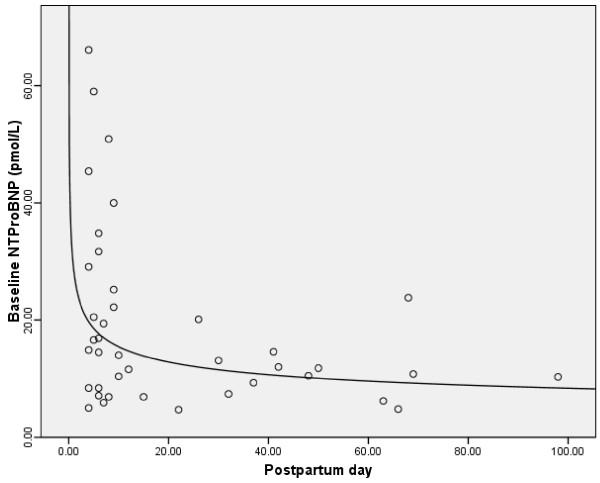
**Scatter plot of baseline NT-proBNP in relation to the postpartum day of sample collection**.

The baseline characteristics of nulliparous and lactating women are shown in Table 1. Subjects were generally healthy and none suffered from diabetes mellitus, hypertension or heart disease. Baseline serum concentrations of 25(OH)D were low (<50 nmol/L) in all subjects except in one nulliparous and one lactating woman and they correlated negatively with PTH (r = -0.1, p = 0.6 in nulliparous women and r = -0.5, p < 0.001 in lactating women). In nulliparous women, NT-proBNP values were undetectable in more than half (51%) of the subjects. In lactating women, NT-proBNP levels were within the normal range (Table 2) but they dropped significantly with increasing postpartum days until they reached a plateau at ~14 days postpartum (Fig 1). There were no statistically significant correlations between NT-proBNP and either 25(OH)D (r = 0.01, p = 0.9) or PTH (r = -0.1, p = 0.4) in nulliparous women. There were also no statistically significant correlations between 25(OH)D and either NT-proBNP (r = 0.003, p = 1.0) or PRA (r = -0.1, p = 0.5) or between PTH and either NT-proBNP (r = 0.1, p = 0.5) or PRA (r = -0.1, p = 0.4) in lactating women.

**Table 1:** Baseline characteristics of study subjects

**Characteristic**	**Nulliparous**	**Lactating**
	n = 63	n = 53

Ethnicity^1^		
UAE (%)	98.4 [62]	15.1 [8]
Other Arab (%)	1.6 [1]	67.9 [36]
South Asian (%)	0 [0]	17.0 [9]
Age (y)^2^	24.0 ± 0.6	29.8 ± 0.9
Weight (kg)^2^	63.7 ± 2.9	72.2 ± 1.7
BMI (kg/m^2^)^2^	24.2 ± 0.7	28.4 ± 0.6
Systolic Bp (mm Hg)^2^	108 ± 1	120 ± 1
Diastolic Bp (mm Hg)^2^	70 ± 1	76 ± 1
Parity^3^	0	3.0
Health^3,4^	1	1
Sunlight exposure (min/day)^2^	5.6 ± 1.8	0.8 ± 0.5
Multivitamin use (%)^1^	3.2 [2]	49 [26]
Vitamin D intake (mcg/day)^2^	3.7 ± 0.3	4.5 ± 0.4
Calcium intake (g/day)^2^	0.5 ± 0.05	0.6 ± 0.05

^1 ^*n *in brackets;

^2 ^Mean ± SEM;

^3 ^Median;

^4 ^Self reported general health status (1–5): excellent, very good, good, fair, poor.

**Table 2:** Baseline biochemical variables in nulliparous and lactating women^1^

**Group**	**Nulliparous women (n = 63)**	**Lactating women (n = 53)**
Serum 25OHD (nmol/L)	19.0 ± 1.4	26.6 ± 1.4
Serum PTH (pmol/L)	7.3 ± 0.4	4.6 ± 0.4
Serum calcium (mmol/L)	2.3 ± 0.01	2.4 ± 0.01
NTproBNp (pmol/L)	1.6 ± 0.3	19.2 ± 2.2
PRA (nmol/L/hr)	-----	2.0 ± 0.1

^1 ^Mean ± SEM;

25(OH)D, 25-hydroxyvitamin D; PTH, parathyroid hormone; NT-proBNP, N-terminal pro B-type natriuretic peptide; PRA, plasma renin activity.

### Follow-up data

**Figure 2:**
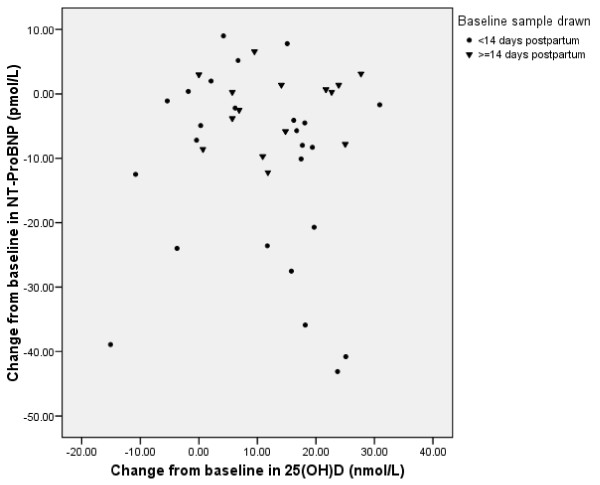
**Correlation between the changes from baseline in 25(OH)D and NT-proBNP by day of collected baseline sample (<14 or ≥14 days postpartum)**.

Of the 58 lactating women who completed 2 months of the study, sufficient stored plasma was available in 53 subjects. Twenty-six women were allocated to the daily and 27 were allocated to the monthly regimen. No significant differences in baseline characteristics were found among subjects based on the allocated regimen or availability of sufficient stored samples (data not shown). As expected, mean concentration of 25(OH)D increased significantly (p < 0.001) over the 2 month period of vitamin D supplementation from 26.6 ± 1.5 to 39.0 ± 1.7 nmol/L (Table 3). Similarly, mean serum PTH concentration decreased but not statistically significantly (Table 3). Over the same time period, plasma levels of NT-proBNP declined in 33 of 46 women, and overall substantially (by 26%) and significantly (p < 0.001) by 9.1 ± 2.0 pmol/L whilst PRA fell in 32 of 53 women, but overall only slightly (by 0.32 ± 0.17 nmol/L/hr) and not statistically significantly (p = 0.064). The above changes were not significantly different between the daily and intermittent supplementation regimen groups (Table 3). Overall, there were no significant changes in mean serum calcium concentrations, blood pressure, and weight. There were no significant correlations between the changes from baseline in 25(OH)D and NT-proBNP (r = -0.04, p = 0.8) whether the baseline sample was drawn <14 or ≥ 14 days postpartum (r = -0.3, p = 0.2 and r = 0.1, p = 0.6; respectively) (Fig 2). In the 10 (18.9%) women who achieved 25(OH)D concentrations of ≥ 50 nmol/L at 2 months, the changes from baseline in 25(OH)D and NT-proBNP also showed no significant correlation (r = 0.5, p = 0.2). There were also no significant correlations between the changes from baseline in 25(OH)D and PRA (r = -0.04, p = 0.8) or between the changes from baseline in PTH and either NT-proBNP (r = -0.02, p = 0.9) or PRA (r = -0.1; p = 0.5).

**Table 3:** Change from baseline in biochemical and clinical variables of lactating women by type of vitamin D supplementation regimen^1^

**Variable**	**Daily regimen**		**Monthly regimen**		**Total**	
	Change	p value	Change	p value	Change	p value^2^

25OHD (nmol/L)	13.9 ± 2.6	<0.001	10.9 ± 1.8	<0.001	12.3 ± 1.6	<0.001
PTH (pmol/L)	0.1 ± 0.4	0.9	-1.0 ± 0.7	0.2	-0.4 ± 0.4	0.3
Calcium mmol/L	-0.09 ± 0.04	0.04	-0.001 ± 0.03	1.0	-0.04 ± 0.02	0.1
NTproBNp (pmol/L)	-10.0 ± 3.0	0.003	-8.2 ± 3.0	0.008	-9.1 ± 2.0	<0.001
PRA (nmol/L/hr)	-0.4 ± 0.2	0.1	-0.2 ± 0.2	0.4	-0.3 ± 0.2	0.06
SBP (mm Hg)	-3.5 ± 2.6	0.2	-0.6 ± 1.8	0.7	-2.0 ± 1.5	0.2
DBP (mm Hg)	-4.4 ± 1.4	0.005	1.0 ± 1.9	0.6	-1.6 ± 1.2	0.2
Weight (kg)	-0.2 ± 0.6	0.7	-0.4 ± 0.6	0.5	-0.3 ± 0.4	0.5

^1 ^Mean ± SEM;

^2 ^*P *value by paired two-tailed *t*-tests.

25(OH)D, 25-hydroxyvitamin D; PTH, parathyroid hormone; NT-proBNP, N-terminal pro B-type natriuretic peptide; PRA, plasma rennin activity; SBP systolic blood pressure; DBP, diastolic blood pressure.

## Discussion

Vitamin D deficiency may adversely affect cardiac function [6-8,20] and vitamin D administration under these conditions may be beneficial [21]. Park and colleagues reported that calcitriol, administered intravenously twice weekly over 15 weeks, reduced left ventricular mass and suppressed circulating levels of renin, angiotensin II and ANP in patients on chronic hemodialysis with secondary hyperparathyroidism [22]. Similar findings of regression of cardiac hypertrophy (together with reduced QT dispersion) during calcitriol administration in a similar cohort of patients were reported subsequently by the same group [23]. Other investigators also found that vitamin D administration for patients with congestive heart failure improved pro-inflammatory and anti-inflammatory cytokine levels but there was no significant decline in NT-proBNP levels [21]. Our results show no significant correlations between baseline 25(OH)D concentrations and NT-proBNP levels in vitamin D deficient nulliparous women or between baseline 25(OH)D concentrations and NT-proBNP and PRA in vitamin D deficient lactating women. Although vitamin D administration over a 2-month period in lactating women was associated with a statistically significant decline in NT-proBNP levels and a non-statistically significant decline in PRA, there were no significant correlations between the change from baseline in 25(OH)D concentrations or PTH with the change from baseline in NT-proBNP and PRA levels. This suggests that the decline we observed in NT-proBNP is unlikely related to vitamin D administration but is probably related to other factors such as postpartum blood volume changes. Pregnancy represents a state of physiologic volume expansion as maternal blood volume increases ~40%–45% above non-pregnancy volumes [24]. By 1 week after delivery, the blood volume returns nearly to its non-pregnancy value [25]. NT-proBNP levels, on the other hand, increase by 2-fold within the first 28 hours after delivery suggesting a role in postpartum diuresis [26]. Our results suggest that NT-proBNP levels drop quickly thereafter until they reach a plateau at ~14 days postpartum.

Our data also confirm previous studies showing a high prevalence of severe vitamin D deficiency among women in the Middle East due to sunshine deprivation and inadequate vitamin D intake [14,15,27]. Our current study did not evaluate seasonal changes in serum 25(OH)D concentrations but our previous studies showed no significant seasonal variation in 25(OH)D concentrations between September and February in the UAE, where there is abundant sunshine year-round [15]. In addition, vitamin D fortification of food is not mandatory in many Middle Eastern countries, and the current dietary intake of vitamin D is relatively low [15]. Vitamin D_2 _supplementation with 2000 IU daily or 60,000 IU monthly for 2 months in this study increased serum 25(OH)D concentrations significantly but these concentrations reached an acceptable level (≥ 50 nmol/L) in only a small proportion of studied women [14]. Although the mean increment observed in 25(OH)D concentration in our study was slightly higher than that reported by other investigators [28] (0.4 nmol/L per 100 IU of vitamin D_2_) it remained lower than that reported for equimolar doses of vitamin D_3 _[29-31]. This could be related to greater potency of vitamin D_3 _compared to vitamin D_2 _[32,33] although this has been recently questioned [34].

Our data must be viewed with appropriate caution. First, neither PRA nor NT-proBNP was elevated at baseline, making physiological significance an unresolved issue. Second, we had no time-matched control women (not receiving vitamin D) and serum 25(OH)D concentrations at 2 months remained below the optimal level of 75 nmol/L in all subjects studied. Additionally, pre- and post-treatment assessments by cardiac echocardiography were unfortunately not performed as that would have provided a useful correlate for the NT-proBNP measurements.

## Conclusion

We found no significant correlations between 25(OH)D or PTH with NT-proBNP and PRA in vitamin D deficient women. There were also no significant correlations between the change from baseline in 25(OH)D concentrations or PTH with the change from baseline in NT-proBNP and PRA levels following vitamin D administration over a 2-month period. Further information is required to clarify the effects of vitamin D administration on cardiac structure and function and prevention of cardiovascular disease.

## Competing interests

The authors declare that they have no competing interests.

## Authors' contributions

HS was responsible for overall design, administration and coordination of the project, analysis of results, and writing the manuscript. MGN organised measurements of NT-proBNP and PRA, and participated in analysis of results and writing the manuscript. CF performed the statistical analysis and participated in analysis of results and writing the manuscript. SB and JY were responsible for sample preparation and biochemical analyses. All authors read and approved the final manuscript.

## Pre-publication history

The pre-publication history for this paper can be accessed here:



## References

[B1] Holick MF (2004). Vitamin D: Importance in the prevention of cancers, type 1 diabetes, heart disease, and osteoporosis. Am J Clin Nutr.

[B2] Chiu K, Chu A, Go V, Soad M (2004). Hypovitaminosis D is associated with insulin resistance and beta cell dysfunction. Am J Clin Nutr.

[B3] Pittas AG, Dawson-Hughes B, Li T, Van Dam RM, Willett WC, Manson JE, Hu FB (2006). Vitamin D and calcium intake in relation to type 2 diabetes in women. Diabetes Care.

[B4] Forman JP, Giovannucci E, Holmes MD, Bischoff-Ferrari HA, Tworoger SS, Willett WC, Curhan GC (2007). Plasma 25-hydroxyvitamin D levels and risk of incident hypertension. Hypertension.

[B5] Wang TJ, Pencina MJ, Booth SL, Jacques PF, Ingelsson E, Lanier K, Benjamin EJ, D'Agostino RB, Wolf M, Vasan RS (2008). Vitamin D deficiency and risk of cardiovascular disease. Circulation.

[B6] Zittermann A, Schleithoff SS, Koerfer R (2005). Putting cardiovascular disease and vitamin D insufficiency into perspective. Br J Nutr.

[B7] Zittermann A, Schleithoff SS, Tenderich G, Berthold HK, Körfer R, Stehle P (2003). Low vitamin D status: a contributing factor in the pathogenesis of congestive heart failure?. J Am Coll Cardiol.

[B8] Dobnig H, Pilz S, Scharnagl H, Renner W, Seelhorst U, Wellnitz B, Kinkeldei J, Boehm BO, Weihrauch G, Maerz W (2008). Independent association of low serum 25-hydroxyvitamin D and 1,25-dihydroxyvitamin D levels with all-cause and cardiovascular mortality. Arch Intern Med.

[B9] Li YC, Kong J, Wei M, Chen ZF, Liu SQ, Cao LP (2002). 1,25-dihydroxyvitamin D_3 _is a negative endocrine regulator of the renin-angiotensin system. J Clin Invest.

[B10] Xiang W, Kong J, Chen S, Cao LP, Qiao G, Zheng W, Liu W, Li X, Gardner DG, Li YC (2005). Cardiac hypertrophy in vitamin D receptor knockout mice: role of the systemic and cardiac renin-angiotensin systems. Am J Physiol Endocrinol Metab.

[B11] Li YC, Feldman D, Pike JW, Glorieux FH (2005). Vitamin D and the renin-angiotensin system. Vitamin D.

[B12] Perkovic V, Hewitson TD, Kelynack KJ, Martic M, Tait MG, Becker GJ (2003). Parathyroid hormone has a prosclerotic effect on vascular smooth muscle cells. Kidney Blood Press Res.

[B13] Bidmon H-J, Gutkowska J, Murakami R, Stumpf WE (1991). Vitamin D receptors in heart: effects on atrial natriuretic factor. Experientia.

[B14] Saadi HF, Dawodu A, Afandi BO, Zayed R, Benedict S, Nagelkerke N (2007). Efficacy of daily and monthly high-dose calciferol in vitamin D deficient nulliparous and lactating women. Am J Clin Nutr.

[B15] Saadi HF, Nagelkerke N, Benedict S, Qazaq HS, Zilahi E, Mohamadiyeh MK, Al-Suhaili AI (2006). Predictors and relationships of serum 25 hydroxyvitamin D concentration with bone turnover markers, bone mineral density, and vitamin D receptor genotype in Emirati women. Bone.

[B16] Saadi H, Kazzam E, Ghurbana BA, Nicholls MG (2006). Hypothesis: correction of low vitamin D status among Arab women will prevent heart failure and improve cardiac function in established heart failure. Eur J Heart Fail.

[B17] Richards AM, Doughty R, Nicholls MG, MacMahon S, Sharpe N, Murphy J, Espiner EA, Frampton C, Yandle TG, Australia-New Zealand Heart Failure Group (2001). Plasma N-terminal pro-brain natriuretic peptide and adrenomedullin: prognostic utility and prediction of benefit from carvedilol in chronic ischemic left ventricular dysfunction. J Am Coll Cardiol.

[B18] Hunt PJ, Richards AM, Nicholls MG, Yandle TG, Doughty RN, Espiner EA (1997). Immunoreactive amino-terminal pro-brain natriuretic peptide (NT-PROBNP): a new marker of cardiac impairment. Clin Endocrinol (Oxf).

[B19] Obineche EN, Saadi H, Benedict S, Pathan JY, Frampton CM, Nicholls MG (2008). Inter-relationships between B-type natriuretic peptides and Vitamin D in patients on maintenance peritoneal dialysis. Peritoneal Dialysis International.

[B20] Zittermann A, Schleithoff SS, Götting C, Dronow O, Fuchs U, Kuhn J, Kleesiek K, Tenderich G, Koerfer R (2008). Poor outcome in end-stage heart failure patients with low circulating calcitriol levels. Eur J Heart Fail.

[B21] Schleithoff SS, Zittermann A, Tenderich G, Berthold HK, Stehle P, Koerfer R (2006). Vitamin D supplementation improves cytokine profiles in patients with congestive heart failure: a double-blind, randomized, placebo-controlled trial. Am J Clin Nutr.

[B22] Park CW, Oh YS, Shin YS, Kim CM, Kim YS, Kim SY, Choi EJ, Chang YS, Bang BK (1999). Intravenous calcitriol regresses myocardial hypertrophy in hemodialysis patients with secondary hyperparathyroidism. Am J Kidney Dis.

[B23] Kim HW, Park CW, Shin YS, Kim YS, Shin SJ, Kim YS, Choi EJ, Chang YS, Bang BK (2006). Calcitriol regresses cardiac hypertrophy and QT dispersion in secondary hyperparathyroidism on hemodialysis. Nephron.

[B24] Whittaker PG, Macphail S, Lind T (1996). Serial hematologic changes and pregnancy outcome. Obstet Gynecol.

[B25] Cunningham FG, Gant NF, Leveno KJ, Gilstrap LC, Hauth JC, Wenstrom KD (2001). The puerperium. Williams Obstetrics.

[B26] Lev-Sagie A, Bar-Oz B, Salpeter L, Hochner-Celnikier D, Arad I, Nir A (2005). Plasma concentrations of N-terminal Pro-B-Type natriuretic peptide in pregnant women near labor and during early puerperium. Clin Chem.

[B27] Dawodu A, Absood G, Patel M, Agarwal M, Ezimokhai M, Abdulrazzaq Y, Khalayli G (1998). Biosocial factors affecting vitamin D status of women of child-bearing age in the United Arab Emirates. J Biosoc Sci.

[B28] Cooper L, Clifton-Bligh PB, Nery ML, Figtree G, Twigg S, Hibbert E, Robinson BG (2003). Vitamin D supplementation and bone mineral density in early postmenopausal women. Am J Clin Nutr.

[B29] Veith R, Feldman D, Pike JW, Glorieux FH (2005). The pharmacology of vitamin D, including fortification strategies. Vitamin D.

[B30] Barger-Lux MJ, Heaney RP, Dowell S, Chen TC, Holick MF (1998). Vitamin D and its major metabolites: serum levels after graded oral dosing in healthy men. Osteoporos Int.

[B31] Heaney RP, Davies KM, Chen TC, Holick MF, Barger-Lux MJ (2003). Human serum 25-hydroxycholecalciferol response to extended oral dosing with cholecalciferol. Am J Clin Nutr.

[B32] Trang HM, Cole DE, Rubin LA, Pierratos A, Siu S, Vieth R (1998). Evidence that vitamin D3 increases serum 25-hydroxyvitamin D more efficiently than does vitamin D2. Am J Clin Nutr.

[B33] Armas LA, Hollis BW, Heaney RP (2004). Vitamin D2 is much less effective than vitamin D3 in humans. J Clin Endocrinol Metab.

[B34] Holick MF, Biancuzzo RM, Chen TC, Klein EK, Young A, Bibuld D, Reitz R, Salameh W, Ameri A, Tannenbaum AD (2008). Vitamin D2 is as effective as vitamin D3 in maintaining circulating concentrations of 25-hydroxyvitamin D. J Clin Endocrinol Metab.

